# Repurposing Azithromycin and Rifampicin Against Gram-Negative Pathogens by Combination With Peptidomimetics

**DOI:** 10.3389/fcimb.2019.00236

**Published:** 2019-07-02

**Authors:** Kristin R. Baker, Bimal Jana, Anna Mette Hansen, Hanne Mørck Nielsen, Henrik Franzyk, Luca Guardabassi

**Affiliations:** ^1^Department of Veterinary and Animal Sciences, Faculty of Health and Medical Sciences, University of Copenhagen, Frederiksberg, Denmark; ^2^Department of Biomedical Sciences, Ross University School of Veterinary Medicine, Basseterre, Saint Kitts and Nevis; ^3^Department of Drug Design and Pharmacology, Faculty of Health and Medical Sciences, University of Copenhagen, Copenhagen, Denmark; ^4^Department of Pharmacy, Faculty of Health and Medical Sciences, University of Copenhagen, Copenhagen, Denmark; ^5^Department of Pathobiology and Population Sciences, Royal Veterinary College, Hatfield, United Kingdom

**Keywords:** peptidomimetic, antibiotic potentiation, multidrug resistance, synergy, Gram-negative, antibiotic adjuvant

## Abstract

Synthetic peptidomimetics may be designed to mimic functions of antimicrobial peptides, including potentiation of antibiotics, yet possessing improved pharmacological properties. Pairwise screening of 42 synthetic peptidomimetics combined with the antibiotics azithromycin and rifampicin in multidrug-resistant (MDR) *Escherichia coli* ST131 and *Klebsiella pneumoniae* ST258 led to identification of two subclasses of α-peptide/β-peptoid hybrids that display synergy with azithromycin and rifampicin (fractional inhibitory concentration indexes of 0.03–0.38). Further screening of the best three peptidomimetics in combination with a panel of 21 additional antibiotics led to identification of peptidomimetics that potentiated ticarcillin/clavulanate and erythromycin against *E. coli*, and clindamycin against *K. pneumoniae*. The study of six peptidomimetics was extended to *Pseudomonas aeruginosa*, confirming synergy with antibiotics for five of them. The most promising compound, H-(Lys-βNPhe)_8_-NH_2_, exerted only a minor effect on the viability of mammalian cells (EC_50_ ≥ 124–210 μM), and thus exhibited the highest selectivity toward bacteria. This compound also synergized with rifampicin and azithromycin at sub-micromolar concentrations (0.25–0.5 μM), thereby inducing susceptibility to these antibiotics at clinically relevant concentrations in clinical MDR isolates. This peptidomimetic lead and its analogs constitute promising candidates for efficient repurposing of rifampicin and azithromycin against Gram-negative pathogens.

## Introduction

Traditional antimicrobial therapies have become ineffective in treating infections caused by multidrug-resistant (MDR) Gram-negative pathogens. Thus, new therapeutic strategies are required to manage these infections (Doi et al., 2017; World Health Organisation, 2017). Emergence of resistance to mono-drug therapies has drawn attention to combination therapies with new agents that limit resistance development or overcome resistance mechanisms (Kalan and Wright, 2011; Brown, 2015; Ribeiro et al., 2015; Brown and Wright, 2016; Melander and Melander, 2017; Singh et al., 2017; Moon and Huang, 2018). In this regard, co-application of certain peptides has been found to constitute a promising means for overcoming intrinsic resistance to certain classes of antimicrobials (e.g., macrolides, lincosamides and rifamycins) in Gram-negative pathogens due to their ability to increase the permeability of the outer membrane (Gill et al., 2015; Baker et al., 2018).

Despite the potential utility of peptides as potentiators of antibiotics, their clinical use is hampered by unfavorable pharmacological properties. In particular, many peptides also affect the viability of mammalian cells and possess inherently low stability toward proteolytic degradation, which (particularly for linear peptides) represents a major obstacle for their development into drugs (Ghosh and Haldar, 2015; Mojsoska and Jenssen, 2015; Molchanova et al., 2017a). While most naturally occurring peptides consist exclusively of α-amino acid residues, peptidomimetics incorporate unnatural amino acids or mimics thereof, resulting in alternative backbones that resist enzymatic cleavage (Molchanova et al., 2017a). In addition, end modifications (Jahnsen et al., 2015) may broaden the chemical space covered. These features of peptidomimetics enable design of compounds with fine-tuned physicochemical properties that confer enhanced antibacterial activity as well as an improved pharmacological profile (Méndez-Samperio, 2014). Moreover, certain peptidomimetics have proved capable of potentiating traditional antibiotics (Renau et al., 2002; Goldberg et al., 2013; Jammal et al., 2015; Lainson et al., 2017), suggesting a possible role in combination therapy. Despite displaying several features that are superior to those of peptides, their therapeutic potential relies on technological advances to enable reduced production cost, improved bioavailability, and minimized toxicity toward host cells (Molchanova et al., 2017a). Nonetheless, recent advances in synthesis of peptidomimetics has enabled investigation of diverse compound arrays, thus facilitating identification of peptidomimetic antibiotic adjuvants with suitable pharmacological properties.

Benefiting from already available peptidomimetic arrays (Hein-Kristensen et al., 2011; Jahnsen et al., 2012, 2014, 2015; Liu et al., 2013), we performed a search for peptidomimetics capable of circumventing the intrinsically low susceptibility to certain antibiotics in Gram-negative pathogens. To address potential toxicity issues, which are considered the most critical factor currently limiting the therapeutic use of peptidomimetics, the screening process was designed so that only compounds that potentiate antibiotics at sub-micromolar nontoxic concentrations were pursued. Thus, screening of pairwise peptidomimetic-antibiotic combinations led to identification of α-peptide/β-peptoid hybrids capable of inducing susceptibility to azithromycin and rifampicin (below their putative clinical breakpoints) in clinical MDR isolates of *Escherichia coli, K. pneumoniae* and *P. aeruginosa*.

## Materials and Methods

### Bacterial Strains, Antibiotics and Media

Bacterial strains used in this study comprise MDR clinical isolates of *E. coli* ST131 (Cerquetti et al., 2010) and *K. pneumoniae* ST258 (Jana et al., 2017), and American type culture collection (ATCC) strains: ATCC 25922 (*E. coli*), ATCC 13883 (*K. pneumoniae*), and ATCC 27853 (*P. aeruginosa*). Additional clinical isolates of *E. coli, K. pneumoniae*, and *P. aeruginosa* resistant to β-lactams (including carbapenems and cephalosporins) were provided by Laurent Poirel, Université de Fribourg, Switzerland. The following growth media was routinely used for bacterial culturing: Luria-Burtani (LB), cation-adjusted Mueller-Hinton agar (MHA) and broth (MHB II). All assays were performed in cation-adjusted MHA or MHB II. Antibiotics were purchased from Sigma-Aldrich and the peptidomimetic collection (Table S1) was curated and provided by Henrik Franzyk. This collection contained peptoids, α-peptide/β-peptoid hybrids, and a variety of end-group modifications, thereby representing a wide range of physicochemical and structural properties, including varied cationic character, length and hydrophobicity. Stock solutions were prepared by dissolving the compounds in deionized water, while test solutions were obtained by further dilution with MHB II medium.

### Peptidomimetic Synthesis

α-Peptide/β-peptoid peptidomimetics were synthesized on a Rink amide resin (loading: 0.5–0.7 mmol/g; 0.05–0.1 mmol scale) in Teflon reactors (10 mL) by standard Fmoc-based solid-phase synthesis using the appropriate dimeric building blocks (Bonke et al., 2008; Jahnsen et al., 2014). Fmoc deprotection was performed with excess 20% piperidine in DMF (2 × 10 min, each time with 5 mL; shaking at room temperature). After Fmoc deprotection (and after coupling as well) the resin was washed with DMF, MeOH, and DCM (each 3 × 3 min with 5 mL). Coupling was performed with the appropriate building block (2.0 equiv. for loading, 2.5 equiv for the first two elongations, and 3.0 equiv. for subsequent elongations) and PyBOP/DIPEA (1:2 equiv. relative to the building block) in DMF (2–3 mL) for >2 h under shaking at room temperature. Capping was applied after the fourth coupling via treatment with Ac_2_O–DIPEA–NMP 1:2:3 (5 mL, 10 min at room temperature). Final Fmoc deprotection was followed by attachment of any N-terminal end group: the carboxylic acid (5 equiv.) corresponding to the desired moiety was added under conditions identical to those applied for the above coupling procedure used for the dimeric building blocks. Cleavage and simultaneous side chain deprotection: Excess TFA-CH_2_Cl_2_ 90:10 (2 × 30 min under shaking at room temperature). The filtrate was collected and the resin was eluted with CH_2_Cl_2_ (2 mL). The combined filtrates were concentrated in vacuo, and co-evaporated three times with toluene. The crude products were purified by using preparative HPLC, and the resulting pure fractions were freeze-dried as previously described (Jahnsen et al., 2012, 2015; Skovbakke et al., 2015; Molchanova et al., 2017b).

### Antimicrobial Susceptibility Testing

Minimal inhibitory concentrations (MICs) of peptidomimetics, antibiotics and their combinations were determined by broth microdilution according to the Clinical Laboratory Standards Institute performance standards (Clinical and Laboratory Standards Institute, 2013). Based on previous studies indicating that among a range of tested antibiotics belonging to several classes azithromycin and rifampicin displayed a high propensity to synergize with peptides, and hence these antibiotics were chosen for the initial screening of peptidomimetics to identify possible antibiotic adjuvants (Jammal et al., 2015; Baker et al., 2016, 2018; Corbett et al., 2017; Lyu et al., 2017; Wu et al., 2017; Domalaon et al., 2018a,b; Yang et al., 2018). Here, a sub-MIC concentration of 0.5 μM peptidomimetic was tested in combination with 0.5 μg/mL rifampicin or 1 μg/mL azithromycin in 96-well plates, followed by inoculum addition according to CLSI guidelines. At these concentrations, control wells containing either peptidomimetic or antibiotic individually did not inhibit growth. Since rifampicin and azithromycin mainly are used to treat infections with Gram-positive pathogens only CLSI clinical breakpoints were available for such bacteria. Hence the clinically relevant test concentrations were selected based on the CLSI susceptibility breakpoints for *Staphylococcus* species (Clinical and Laboratory Standards Institute, 2017): rifampicin (1 μg/mL) and azithromycin (2 μg/mL). Potentiation of additional antibiotics by peptidomimetics was determined by using commercial 96-well plates containing varied concentrations of standard antibiotics (COMPAN1F; Trek Diagnostic Systems Inc.). The peptidomimetics were added immediately prior to inoculation with pathogen, and then MICs were determined as above. The MICs of peptidomimetic/antibiotic combinations were determined according to the method above, and the concentration ratios were selected based upon checkerboard results with 2:1 and 1:4 ratio of peptidomimetic with rifampicin or azithromycin, respectively. Minimal bactericidal concentrations (MBCs) were determined by using growth-curve assay plates. MIC plates were prepared as above, and then the plates were incubated at 37°C with continuous shaking. Growth was recorded at 10 min intervals as optical density (OD) at 600 nm during 24 h. At 24 h, for wells without growth, a sample was diluted 10-fold in sterile saline (0.9% sodium chloride) and plated onto MHA, following CLSI guidelines (Clinical and Laboratory Standards Institute, 1999).

### Checkerboard Synergy Assay

The potential synergistic effects in peptidomimetic-antibiotic combinations were assessed by using a two-dimensional checkerboard assay (Garcia, 2010). Briefly, twofold dilutions of peptide and antibiotic were prepared in MBH II media along the X- and Y-axis, respectively, in 96-well plates. The inoculum was added to the plates, and subsequently these were incubated for 18–20 h. All steps for inoculum preparation and broth microdilution were in accordance with the CLSI guidelines (Clinical and Laboratory Standards Institute, 2013). The fractional inhibitory concentration index (FICI) was calculated as [MIC_A(A+B)_/MIC_A(alone)_] + [MIC_B(A+B)_/MIC_B(alone)_], where MIC_A(A+B)_ represents the MIC of antibiotic (A) in combination with peptidomimetic (B), while MIC_B(A+B)_ represents the MIC of peptidomimetic (B) in combination with antibiotic (A). MIC_A(alone)_ and MIC_B(alone)_ represent the MIC of each compound individually. FICI values of ≤0.5 were interpreted as synergy.

### Determination of Cell Viability and Selectivity Indexes for Peptidomimetics

NIH 3T3 and HepG2 cells (both from ATCC, Manassas, VA, USA) were cultured in flat-bottomed 96-well MicroWell™ plates (NUNC, Roskilde, Denmark) at seeding densities of ~23,000 and ~22,000 cells/well, respectively, and then cultured for 22–24 h under standard culturing conditions (37°C, 5% CO_2_, humidified air) to reach 80–90% confluence. NIH 3T3 were cultured in Dulbecco's modified Eagle's medium (DMEM) supplemented with 10% (v/v) newborn-calf serum (NCS) (Gibco, Paisly, UK), while HepG2 were cultured in Eagle's minimum essential medium (EMEM) supplemented with 10% (v/v) fetal bovine serum (FBS, Gibco, Paisly, UK), sodium pyruvate (1 mM), and non-essential amino acids (1%, v/v). Both culturing media were further supplemented with penicillin (100 U/mL), streptomycin (100 μg/mL), and L-glutamine (2 mM).

Effect on cell viability was determined in NIH 3T3 fibroblasts and HepG2 hepatocytes by using the MTS/PMS assay measuring metabolic activity. Briefly, the adhered cells were washed with 37°C Hanks' balanced salt solution (HBSS from Sigma-Aldrich, St. Louis, MO, USA), containing 10 mM HEPES (AppliChem, Darmstadt, Germany), and adjusted to pH 7.4, and were then exposed for 1 h to 100 μL of test compound(s) dissolved in the appropriate culturing medium without serum for each cell line. After exposure the cells were washed with HBSS containing 10 mM HEPES (pH 7.4) and 100 μL of an MTS/PMS solution, consisting of 240 μg/mL MTS (Promega, Madison, WI, USA) and 2.4 μg/mL PMS (SigmaAldrich, Buchs, Switzerland) in HBSS, was added to the cells, which then were incubated for 1 h at 37°C with horizontal shaking under light protection. Absorbance was measured at 492 nm by using a POLARstar OPTIMA plate reader (BMG Labtech, Offenburg, Germany). The relative viability was calculated according to Equation (1) with absorbance values obtained after incubation of cells with test compound; incubation with SDS (0.2%, w/v in medium) defined 100% cell death (Abs_pos_), while the absorbance of cells incubated with medium defined 0% cell death (Abs_neg_).

(1) $$\begin{array}{l} Relative\;viability\left(\%\right)=\frac{\left(Abs_{sample}-Abs_{pos}\right)}{\left(Abs_{neg}-Abs_{pos}\right)}\times 100\% \end{array}$$

EC_50_ values were calculated by using GraphPad Prism 7 (GraphPad Software, La Jolla, CA, USA) via fitting of the relative cell viability to the concentration of the test compound by using Equation (2):

(2) $$\begin{array}{l} Relative\;viability\left(\%\right)=\;\frac{Top-Bottom}{1+10^{\left(LogIC_{50}-Log\left[peptidomimetic\right]\right)\;\times Hill\;slope}} \end{array}$$

With top and bottom representing the mean highest and lowest observed values, respectively. The Hill slope represents a factor for the steepness of the linear part of the curve. In order to prevent erroneous calculation, the top and bottom values were constrained to 100 and 0%, respectively. Data were collected from technical triplicates. The selectivity index was calculated as the average effect on cell viability (EC_50_ values for HepG2 and NIH 3T3 cells) divided by the MIC.

### Time-Kill Assay

Time-kill kinetic assays were performed in MDR *E. coli* ST131, MDR *K. pneumoniae* ST258, and *P. aeruginosa* ATCC 27853. Overnight cultures were subcultured in MHB II and grown to the logarithmic phase, after which ~10^6^ CFU/mL cells were transferred to 15-mL round-bottom Falcon tubes. Bacterial cells were incubated at 37°C with aeration in the presence or absence of antibiotic, peptidomimetic or a combination of these at 2 × MIC (as observed in the growth curves; **Table 6**). At time points 0, 1, 2, 4, 8, and 24 h, 100 μl cells were serially diluted 10-fold in sterile 0.9% NaCl, and then 10 μl aliquots were plated on MHA in triplicate. The CFU/mL from each condition was calculated from counted colonies following 18–24 h incubation at 37°C. The detection limit was ~10^2^ CFU/mL. The CFU/mL of each culture was graphed in GraphPad Prism 7 over time (h), and all graphs represent the average and standard error from two separate experiments (i.e., biological duplicates). Synergy was defined as a ≥2-log_10_ CFU/mL decrease for the cultures exposed to the antibiotic-peptidomimetic combination vs. either compound individually. Bactericidal activity was defined as a ≥3-log_10_ CFU/mL decrease after 24 h incubation.

## Results

### Identification of Two Subclasses of Peptidomimetics That Induce Susceptibility to Rifampicin and Azithromycin in Gram-Negative Pathogens

**Figure 1:**
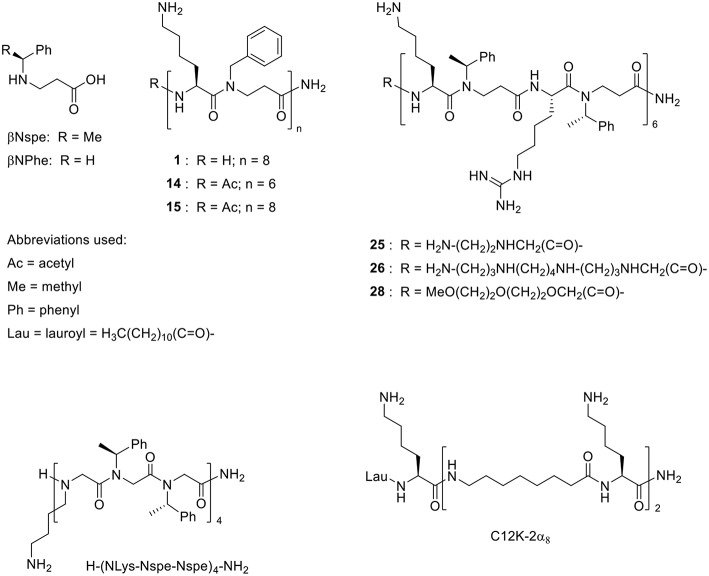
Structures of the α-peptide/β-peptoid hybrids listed in Table 2 and similar compounds that exhibit activity *in vivo* (Zaknoon et al., 2011; Czyzewski et al., 2016, respectively).

Screening of peptidomimetic-antibiotic combinations was performed in order to identify potentiators of rifampicin and azithromycin in MDR *Escherichia coli* sequence type 131 (ST131) and *K. pneumoniae* ST258 epidemic clones. This involved 42 selected diverse peptidomimetics that were combined at 0.5 μM with each antibiotic at concentrations approximating the clinical breakpoints in Gram-positive species (i.e., 0.5 μg/mL and 1 μg/mL, for rifampicin and azithromycin, respectively) (Clinical and Laboratory Standards Institute, 2017). Three peptidomimetics (i.e., **1**, **15**, and **26**) were found to inhibit growth of both species in the presence of rifampicin, while one compound (i.e., **1**) prevented growth in combination with azithromycin (Table 1). These peptidomimetics represent two subclasses of α-peptide/β-peptoid hybrids: subclass I (e.g., **1** and **15**) comprising lysine-based compounds with achiral β-peptoid phenylalanine-like residues, and subclass II (e.g., **26**) displaying both Lys and homoarginine (hArg) as cationic residues together with α-chiral β-peptoid hydrophobic Phe-like residues (Table 2; Figure 1). The effect on cell viability of these three peptidomimetics was tested toward the mammalian cells lines mouse fibroblasts (NIH 3T3) and human hepatocytes (HepG2), which revealed that **1** and **15** exhibited promising cell selectivity (i.e., EC_50_ >100 μM) while peptidomimetic **26** proved to affect cell viability the most with EC_50_ values in the range 50–60 μM (Table 2). These peptidomimetics were further screened for potentiation of additional 21 antibiotics (Table 3) at sub-MIC concentrations of ≤1 μM (Table 2). In presence of peptidomimetics **1**, **15**, or **26**, the MIC of rifampicin (>2 μg/mL) was reduced to below 1 μg/mL in both MDR *E. coli* and *K. pneumoniae*, while the MIC of erythromycin was reduced from >4 μg/mL to ≤2 μg/mL in MDR *E. coli*, confirming the results from the primary screening. In addition, the MIC of clindamycin in MDR *K. pneumoniae* was reduced from >4 μg/mL to 2 μg/mL in presence of 1 μM of **26**, while the MIC of ticarcilllin/clavulanic acid in *E. coli* was reduced from 64 μg/mL to 16 μg/mL in presence of 0.5 μM of **1**. Since this *E. coli* strain was already susceptible to ticarcillin/clavulanic acid, this combination was not studied further. Growth inhibition occurred at concentrations ≥4-fold below the MICs of the peptidomimetic (except for **15** in *E. coli*) and antibiotic alone, indicating that the combinations exerted synergistic effects. Synergy was confirmed by the checkerboard assay, and fractional inhibitory concentration indexes (FICIs) ranged from 0.05 to 0.38 (Table 4). With FICIs ranging from 0.05 to 0.07, the synergistic peptidomimetic-rifampicin interactions in *K. pneumoniae* were especially potent. The peptidomimetic **26**-clindamycin synergistic interaction in *K. pneumoniae* was also potent, however, due to the relatively high effect of **26** on the viability of eukaryotic cells, this combination was not studied further.

**Table 1:** Susceptibility of MDR *E. coli* ST131 and *K. pneumoniae* ST258 to rifampicin (RIF) or azithromycin (AZM) in combination with peptidomimetics^a^.

		Peptidomimetic number (0.5 μM)																																									
**Species**	**ABX**	**1**	**2**	**3**	**4**	**5**	**6**	**7**	**8**	**9**	**10**	**11**	**12**	**13**	**14**	**15**	**16**	**17**	**18**	**19**	**20**	**21**	**22**	**23**	**24**	**25**	**26**	**27**	**28**	**29**	**30**	**31**	**32**	**33**	**34**	**35**	**36**	**37**	**38**	**39**	**40**	**41**	**42**
*E. coli* ST131	RIF^b^	**–**	+	**–**	+	**–**	**–**	+	+	+	**–**	+	+	+	**–**	**–**	+	+	+	+	**–**	**–**	**–**	+	+	**–**	**–**	+	**–**	+	+	+	+	+	+	+	+	**–**	+	+	+	**–**	+
*K. pneumoniae* ST258	(0.5 μg/mL)	**–**	+	+	+	+	+	+	+	+	+	+	+	+	+	**–**	+	+	+	+	+	+	+	+	+	+	**–**	+	+	+	+	+	+	+	+	+	+	+	+	+	+	+	+
*E. coli* ST131	AZM^c^	**–**	+	+	+	**–**	**–**	+	+	+	+	+	+	+	**–**	**–**	+	+	+	**–**	+	+	**–**	+	+	**–**	**–**	+	**–**	+	+	+	+	+	+	+	+	+	+	+	+	**–**	+
*K. pneumoniae* ST258	(1 μg/mL)	**–**	+	+	+	+	+	+	+	+	+	+	+	+	+	+	+	+	+	+	+	+	+	+	+	+	+	+	+	+	+	+	+	+	+	+	+	+	+	+	+	+	+

*Peptidomimetics that were studied further are indicated in bold (see Table 2 for sequences of these)*.

a *Growth (+) and growth inhibition (**–**)*.

b *At 0.5 μg/mL*.

c *At 1 μg/mL*.

**Table 2:** Peptidomimetics studied: sequence, cytotoxicity, and MIC in clinical isolates of *E. coli, K. pneumoniae* and *P. aeruginosa*.

					**Cytotoxicity (μM)**		**MIC (μM) and Selectivity Index (SI^d^; in brackets)**					
**No**.	**Compound**	**MW (g/mol)**	**Charge**	**Length (residues)**	**NIH 3T3 EC_**50**_ ± SD**	**HepG2 EC_**50**_ ± SD**	***E. coli*** **ST131**		***K. pneumoniae*** **ST258**		***P. aeruginosa*** **ATCC 27853**	
**Subclass I**												
**1**	H-(Lys-βNPhe)_8_-NH_2_	3470.44	+9	16	210.60 ± 26.78	123.97 ± 0.81	2	(84)	16	(10)	1	(167)
**14**	Ac-(Lys-βNPhe)_6_-NH_2_	2478.44	+6	12	323.50 ± 45.78	233.53 ± 7.95	4	(70)	64	(4)	16	(17)
**15**	Ac-(Lys-βNPhe)_8_-NH_2_	3286.24	+8	16	216.57 ± 23.12	121.37 ± 0.06	1	(169)	32	(5)	1	(169)
**Subclass II**												
**25**	NDab^a^-Lys-βNspe-hArg-βNspe)_3_-NH_2_	2975.85	+8	13	166.90 ± 5.53	85.01 ± 3.06	2	(63)	8	(16)	8	(63)
**26**	[Spermine-Ac]^b^-(Lys-βNspe-hArg-βNspe)_3_-NH_2_	3346.14	+10	13	53.20 ± 2.94	59.62 ± 0.71	1	(56)	8	(7)	2	(28)
**28**	TODA^c^-(Lys-βNspe-hArg-βNspe)_3_-NH_2_	2807.85	+6	13	217.57 ± 18.05	118.23 ± 2.05	2	(84)	16	(10)	16	(10)

a *NDab, H_2_NCH_2_CH_2_NHCH_2_(C = O)*.

b *Spermine-Ac, H_2_N(CH_2_)_3_NH(CH_2_)_4_NH(CH_2_)_3_NHCH_2_(C = O)*.

c *TODA, H_3_CO(CH_2_)_2_O(CH_2_)_2_O(CH_2_)(C = O)*.

d *SI was calculated as the average of the EC_50_ values for HepG2 and NIH 3T3 cells divided by the MIC*.

**Table 3:** Potentiation of antibiotics by peptidomimetics in MDR *E. coli* ST131 and *K. pneumoniae* ST258; MICs (μg/mL) of antibiotics in presence and absence of peptidomimetics; MICs reduced ≥4-fold in presence of a peptidomimetic are indicated in bold.

**Strain**	***K. pneumoniae*** **ST258**				***E. coli*** **ST131**			
**Peptidomimetic**	**1**	**15**	**26**	**antibiotic alone**	**1**	**15**	**26**	**antibiotic alone**
	**1 μM**	**1 μM**	**1 μM**	**-**	**0.5 μM**	**0.5 μM**	**0.25 μM**	**-**
**β-lactams**								
Ampicillin	>16	>16	>16	>16	>16	>16	>16	>16
Penicillin	>8	>8	>8	>8	>8	>8	>8	>8
Oxacillin (2% NaCl)	>4	>4	>4	>4	>4	>4	>4	>4
Amoxicillin/clavulanic acid (2:1)	>32	>32	>32	>32	8	8	8/4	16
Ticarcillin	>64	>64	>64	>64	>64	>64	ND	>64
Ticarcillin/clavulanic acid (2:1)	>64	>64	>64	>64	**16**	32	ND	64
**Cephalosporins and Carbapenems**								
Cefazolin	>16	>16	>16	>16	>16	>16	>16	>16
Cefovecin	>8	>8	>8	>8	>8	>8	>8	>8
Cefoxitin	>16	>16	>16	>16	>16	4	ND	8
Cefpodoxime	>16	>16	>16	>16	>16	>16	>16	>16
Ceftiofur	>4	>4	>4	>4	>4	>4	ND	>4
Imipenem	>8	>8	>8	>8	≤1	≤1	≤1	≤1
**Aminoglycosides**								
Amikacin	32	32	16	32	>32	8	8	16
Gentamicin	≤1	2	≤1	2	>8	>8	>8	>8
**fluoroquinolone**								
Enrofloxacin	>2	>2	>2	>2	>2	>2	>2	>2
Marbofloxacin	>2	>2	>2	>2	>2	>2	>2	>2
**Miscellaneous**								
Chloramphenicol	>16	>16	>16	>16	≤4	≤4	≤4	≤4
Clindamycin	>4	>4	**2**	>4	4	>4	>4	>4
Doxycycline	≤2	≤2	≤2	4	≤2	≤2	≤2	≤2
Erythromycin	>4	>4	>4	>4	**1**	**2**	**2**	>4
Rifampicin	**≤1**	**≤1**	**≤1**	>2	**≤1**	**≤1**	**≤1**	>2
Trimethoprim/sulfamethoxazole	>38	>38	>38	>38	>38	>38	>38	>38

*Peptidomimetic MICs are reported in Table 2 and as follows: 16, 32, 8, 2, 1 and 1 μM, respective to the order listed in Table 3*.

**Table 4:** Interaction of azithromycin (AZM) and rifampicin (RIF) with peptidomimetics in *E. coli* ST131, *K. pneumoniae* ST258, and *P. aeruginosa* as determined in the checkerboard assay.

**Species**	**Antibiotic (μg/mL)**			**Peptidomimetic (μM)**			**FICI**
	**Agent**	**MIC alone**	**MIC in combination**	**Compound no**.	**MIC alone**	**MIC in combination (Selectivity index)**	
***E. coli*** **ST131**							
	AZM	8	1	**1**	2	0.25 (669)	0.25
			1	**14**	4	0.25 (1114)	0.19
			1	**15**	1	0.13 (1352)	0.25
			0.5	**25**	2	0.25 (504)	0.19
			1	**26**	1	0.25 (226)	0.38
			0.5	**28**	2	0.5 (336)	0.31
	RIF	4	0.06	**1**	2	0.5 (335)	0.27
			0.25	**14**	4	0.25 (1114)	0.13
			0.5	**15**	1	0.13 (1352)	0.25
			0.5	**25**	2	0.25 (504)	0.25
			0.5	**26**	1	0.25 (226)	0.38
			0.13	**28**	2	0.5 (336)	0.28
***K. pneumoniae*** **ST258**							
	AZM	32	4	**1**	16	0.5 (335)	0.16
			4	**14**	64	2 (139)	0.16
			8	**15**	32	0.5 (338)	0.27
			8	**25**	8	0.5 (252)	0.31
			8	**26**	8	0.5 (113)	0.31
			4	**28**	16	2 (84)	0.25
	RIF	16	0.25	**1**	16	0.5 (335)	0.05
			1	**14**	64	1 (279)	0.08
			0.5	**15**	32	0.5 (338)	0.05
			0.06	**25**	8	1 (126)	0.13
			0.06	**26**	8	0.5 (113)	0.07
			0.5	**28**	16	1 (168)	0.09
	CLI	125	4	**26**	8	0.5 (113)	0.09
***P. aeruginosa*** **ATCC 27853**							
	AZM	>128	8	**1**	1	0.25 (669)	0.28
			1	**14**	16	4 (70)	0.25
			2	**15**	1	0.5 (338)	0.51
			4	**25**	8	1 (126)	0.14
			0.5	**26**	2	1 (56)	0.50
			0.5	**28**	16	2 (84)	0.13
	RIF	32	1	**1**	1	0.25 (669)	0.28
			1	**14**	16	4 (70)	0.28
			0.5	**15**	1	0.5 (338)	0.52
			0.06	**25**	8	1 (126)	0.13
			0.06	**26**	2	0.5 (113)	0.25
			0.06	**28**	16	4 (42)	0.25

*Compound numbers are stated in bold*.

To explore additional compounds within the two identified subclasses of peptidomimetics, found to enhance the antibacterial effect of rifampicin and azithromycin, the study was extended to include three additional peptidomimetics, **14** and **25**, **28** (also belonging to subclasses I or II; Table 2). These three peptidomimetics also exhibited an ability to potentiate rifampicin and azithromycin in MDR *E. coli* and *K. pneumoniae* (Table 4). Overall, the degree of synergy was similar to that seen for combinations containing **1**, **15**, and **26**, resulting in potentiation of rifampicin and azithromycin (to reach MICs of ≤1 and ≤8 μg/mL, respectively) in both species. Similar to the subclass I compounds (i.e., **1** and **15**), peptidomimetic **14** displayed the lowest effect on cell viability with EC_50_ values of 323 μM and 234 μM in NIH 3T3 and HepG2, respectively (Table 2). In contrast, peptidomimetic **25** had a noticeably higher effect on cell viability with EC_50_ values in the range 80–170 μM, although somewhat less toxic than the highly cationic peptidomimetic **26**. Among, the subclass II compounds, peptidomimetic **28** had the least effect on cell viability (EC_50_ >100 μM). Overall, the HepG2 liver cell line was more sensitive to the peptidomimetics than the fibroblast cells (i.e., NIH 3T3).

We extended our study to species outside Enterobacteriaceae by including a reference strain of *P. aeruginosa*, which displayed a susceptibility pattern similar to that found for *E. coli* (Table 2). Similarly, synergy was observed with FICIs ranging from 0.13 to 0.5, except for combinations with peptidomimetic **15**, which only displayed a slight potentiation of antibiotic activity (i.e., FICIs >0.5; Table 4). In the presence of sub-MIC concentrations of peptidomimetics **1**, **14**, **15**, **25**, **26**, or **28**, the MICs of azithromycin were reduced 32- to 512-fold, corresponding to MIC values within the range 0.5-8 μg/mL in the synergistic combinations (Table 4). However, the most pronounced enhancement of antibiotic potency was observed when the peptidomimetics were used in combination treatment of *K. pneumoniae*, since the peptidomimetics in this case could be applied in 8- to 64-fold lower concentrations than their MICs as compared to only 2- to 16-fold lower than their MICs in *E. coli* and *P. aeruginosa*.

As a preliminary investigation of the therapeutic potential of the present subclasses of peptidomimetic antibiotic adjuvants, their cell selectivity was estimated as a measure that reflects both antibacterial activity and effect on viability of mammalian cells. Typically, a cell selectivity index (SI) is calculated as the ratio between the averaged EC_50_ values and the MIC of the peptidomimetic in each bacterial species. Peptidomimetics **1** and **15**, belonging to subclass I, proved to be the most selective against *E. coli* and *P. aeruginosa* over mammalian cells with SIs within the range 84–169 (Table 2). When targeting MDR *K. pneumoniae* all compounds displayed poor selectivity, with **25** having a modest SI of 16. As the MICs of the peptidomimetics in combination with antibiotics were lower, the SIs of the combinations with azithromycin or rifampicin were improved, assuming a negligible contribution from the antibiotics to the negative effects on mammalian cell viability, since these were employed in clinically relevant concentrations (Baker et al., 2018). Toward *E. coli*, the most favorable combinations, containing peptidomimetics **14** and **15**, would have approximated SIs >1,000 (Table 4). However, these two peptidomimetics were less selective against *K. pneumoniae* and *P. aeruginosa* with SIs within the range 70–338. While **14** had the highest EC_50_ (Table 2), the concentration of **14** required in synergistic combinations were ≥1 μM and 4 μM, in *K. pneumoniae* and *P. aeruginosa*, respectively, (Table 4). Likewise, compound **15** did not display synergy with any of the antibiotics in *P. aeruginosa* (Table 4). In contrast, peptidomimetic **1** exhibited synergy with both rifampicin and azithromycin at submicromolar concentrations with estimated SIs ≥335 against all three species (Table 4). With an average EC_50_ >150 μM, peptidomimetic **1** was also considered relatively non-toxic in itself. In addition, peptidomimetic **1** was the only compound that exhibited synergy at submicromolar concentrations in combination with antibiotics at clinically relevant concentrations in all three species, and thus this compound was selected for further studies.

### Bacterial Growth and Killing in Presence of Peptidomimetic 1-Antibiotic Combinations

To ascertain that susceptibility of clinical isolates of Enterobacteriaceae and *P. aeruginosa* to combinations of peptidomimetic **1** with rifampicin or azithromycin could be induced, the combinations were tested with respect to MIC against several MDR isolates of each species. Overall, peptidomimetic **1** (at ≤1 μM) induced susceptibility to rifampicin and azithromycin in 83% of the isolates (i.e., in 30 of 36 isolates; Table 5). Notably, most *E. coli* isolates proved susceptible to 0.25–0.5 μg/mL rifampicin and 2–4 μg/mL azithromycin in combination with 0.5–1 μM peptidomimetic **1**. While submicromolar concentrations of **1** in the majority of *K. pneumoniae* isolates were sufficient to induce susceptibility to rifampicin and azithromycin, three isolates required considerably higher concentrations of **1** (i.e., 2–8 μM). By contrast *P. aeruginosa* isolates typically required 1–8 μM of **1** to reduce the MIC of rifampicin and azithromycin to 0.5 μg/mL and 4–8 μg/mL, respectively.

**Table 5:** MICs of Peptidomimetic (PM) **1**-rifampicin and PM **1**-azithromycin combinations in *E. coli, K. pneumoniae*, and *P. aeruginosa* clinical isolates and reference strains.

	**PM 1 / Rifampicin**	**PM 1/ Azithromycin**
**Species**	**MIC (μM/μg mL^**−1**^)**	**MIC (μM/μg mL^**−1**^)**
***E. coli*** **Isolates**		
1	0.5/0.25	0.5/2
2	1/0.5	0.5/2
3	0.5/0.25	0.5/2
4	0.5/0.25	1/4
5	0.5/0.25	1/4
6	1/0.5	1/4
7	0.5/0.25	1/4
8	0.5/0.25	1/4
9	0.5/0.25	0.5/2
10	0.5/0.25	0.5/2
ST131	0.5/0.25	1/4
ATCC 25922	0.5/0.25	0.5/2
***K. pneumoniae*** **Isolates**		
1	0.5/0.25	>4/16
2	8/4	0.5/2
3	0.5/0.25	0.5/2
4	1/0.5	0.5/2
5	0.5/0.25	1/4
6	2/1	0.5/2
7	1/0.5	0.5/2
8	0.5/0.25	0.5/2
9	0.5/0.25	0.5/2
10	4/2	>4/16
ST258	0.5/0.25	4/16
ATCC 13883	0.5/0.25	0.5/2
***P. aeruginosa*** **Isolates**		
1	1/0.5	1/4
2	1/0.5	2/8
3	1/0.5	1/4
4	1/0.5	2/8
5	0.5/0.25	1/4
6	1/0.5	1/4
7	1/0.5	1/4
8	1/0.5	0.5/2
9	1/0.5	1/4
10	4/2	1/4
11	8/4	>4/16
ATCC 27853	0.5/0.25	1/4

To understand the dynamics of the interaction of peptidomimetic **1** with antibiotics, the kinetics of bacterial growth and killing were investigated by growth-curve and time-kill assays. All combinations exhibited synergistic growth inhibition or bactericidal activity in growth-curve and time-kill assays. The growth curves of the combinations corresponded well to the MICs (Table 6) found in static growth assays (Tables 4, 5). In the time-kill assays, the concentrations of tested compounds were 2-fold above the MIC observed in the growth-curve assays, since a 2-fold higher bacterial concentration was used. The tested peptidomimetic **1**-rifampicin combinations were bactericidal in all three species, as at least a 3-log reduction in CFU/mL was reached within the initial 4 h of exposure (**Figures 3A–C**). Similarly, peptidomimetic **1**-azithromycin combinations (**Figures 3D,F**) were bactericidal in *E. coli* and *P. aeruginosa*. However, in *K. pneumoniae* (**Figure 3E**), the combination was bacteriostatic as it only reduced the initial inoculum CFU by ~1.5 log, also reflecting the high MBC, measured at the end of the growth-curve assay (Table 6). Although the MBCs of combinations in *P. aeruginosa* were only 2-fold higher than the MIC (Table 6), in the time-kill assay this species started to recover after 4 h of exposure to the rifampin- and azithromycin-peptidomimetic **1** combinations (**Figures 3C,F**). A similar, but less pronounced, trend was observed for the **1**-rifampicin combination in *E. coli*, for which the CFU concentration had increased at the 24 h time point (**Figure 3A**). In addition, reduced growth was seen for *E. coli* and *P. aeruginosa* in the presence of **1** alone (at concentrations 2-fold below the MIC) within the first 4 h of exposure (**Figures 3A,C,D**).

**Table 6:** MIC and MBC of peptidomimetic (PM) **1** (μM), azithromycin (AZM) and rifampicin (RIF) (μg/mL) and combinations (peptidomimetic + antibiotic) as determined in the growth-curve assay.

	***E. coli*** **ST131**		***K. pneumoniae*** **ST258**		***P. aeruginosa*** **ATTC 27853**	
**Agent**	**MIC**	**MBC**	**MIC**	**MBC**	**MIC**	**MBC**
AZM	8	>16	>32	>32	>128	>128
PM1 + AZM	0.25 + 1	0.5 + 2	0.5 + 4	4 + 32	0.25 + 8	0.5 + 16
RIF	>4	>4	>16	>16	>64	>64
PM1 + RIF	0.25 + 0.31	0.25 + 0.31	0.25 + 0.125	0.25 + 0.125	0.5 + 1	1 + 2
PM1	1	1	8	16–32	1	2

## Discussion

In the present study an array of peptidomimetics was screened in order to identify possible potentiators of antibiotics against MDR Gram-negative pathogens. In particular, the aim was to repurpose antibiotics typically used against Gram-positive pathogens. This enabled identification of a lead compound, peptidomimetic **1**, which exhibited high cell selectivity and displayed synergy with rifampicin and azithromycin at submicromolar, sub-MIC concentrations. Moreover, this compound was found capable of inducing susceptibility to antibiotics in MDR strains and clinical isolates of *E. coli, K. pneumoniae* and *P. aeruginosa*. Initial characterization of five additional peptidomimetics with similar structural composition also revealed potent antibacterial synergy with rifampicin and azithromycin, suggesting that the α-peptide/β-peptoid backbone constitutes a promising template for design of potentiators of antibiotics that Gram-negative pathogens are intrinsically resistant to.

**Figure 2:**
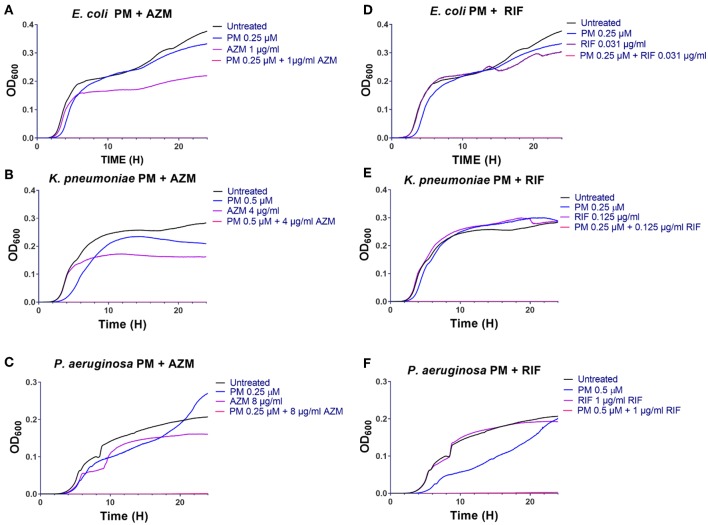
Peptidomimetic **1** potentiates activity of azithromycin and rifampicin in growth-curve assays **(A–F)**. Growth of *E. coli* ST131 **(A,D)**, *K. pneumoniae* ST258 **(B,E)** and *P. aeruginosa* ATCC 27853 **(C,F)** in absence or presence of antibiotic, peptidomimetic (PM) **1** or their combinations were recorded at regular intervals by measuring the optical density (OD) of each culture at 600 nm, and then these data were graphed over time (h). Initial bacterial concentration was ~5 ×10^5^ CFU/mL. The MIC values of each compound are listed in Table 6.

**Figure 3:**
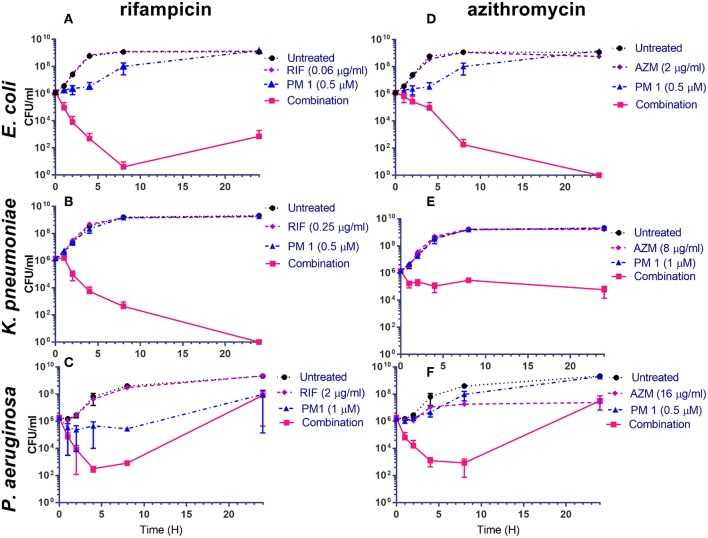
Peptidomimetic **1**-combinations enhance bactericidal activity **(A–F)**. Time-kill kinetics for *E. coli* ST131 **(A,D)**, *K. pneumoniae* ST258 **(B,E)** and *P. aeruginosa* ATTC 27853 **(C,F)** exposed to peptidomimetic (PM) **1**, azithromycin (AZM), rifampicin (RIF), peptidomimetic-antibiotic combinations or without treatment (controls); CFU/mL data are graphed at time points 0, 1, 2, 4, 8, and 24 h of exposure. Initial bacterial concentration was ~10^6^ CFU/mL. The MBC values of each compound are listed in Table 6.

The structural features of the peptidomimetics, identified as antibiotic adjuvants, have been examined in previous studies, focusing on the structure-activity relationships as potential directly antimicrobial agents (Jahnsen et al., 2014; Molchanova et al., 2017b); yet the present work constitutes the first report on their potential for synergy with traditional antibiotics. While the effect of human serum albumin and plasma on the antibiotic adjuvant activity was not addressed in this study, we have in previous work found that the antibacterial activity of similar peptidomimetics (belonging to the same peptidomimetic class) is independent of the presence of physiological concentration human serum albumin, while test in 25% plasma in fact increased their antimicrobial potency (Hein-Kristensen et al., 2013; Citterio et al., 2016). Likewise, the mechanism of synergy has not been investigated in the present study, but this type of peptidomimetics was previously found to exhibit bactericidal activity via membrane permeabilization (Hein-Kristensen et al., 2011), suggesting that at low concentrations these compounds may cause non-lethal membrane alterations that facilitate enhanced influx of antibiotics. The transient growth inhibition induced by these compounds at sub-MIC concentrations in growth-curve and time-kill assays (Figures 2, 3) supports this hypothesis. The six studied peptidomimetics range in length from 12 to 16 residues and display N-terminal end groups (Table 2; Figure 1). Subclass I peptidomimetics display a 1:1 ratio of alternating Lys residues and βNPhe peptoid units as well as either no end group or an acetyl group at the N-terminus (Table 1). Compound **1** had previously been found to be active against the Gram-negative *E. coli, P. aeruginosa, Acinetobacter baumannii* and *Salmonella Typhimurium* as well as the Gram-positive *Staphylococcus pseudintermedius* (Molchanova et al., 2017b), while **15** had proved antibacterial against *E. coli* (Jahnsen et al., 2012). Interestingly, compound **14** at 1 μM has been shown to block the pro-inflammatory effect of LPS (Skovbakke et al., 2015), thereby implying that LPS released from lysed bacteria might constitute a less critical issue when applying this compound as an antibiotic adjuvant.

In accordance with the high structural similarity between **1** and **15**, these analogs exhibited almost equipotent antibacterial, cytotoxic activity and potentiation of antibiotics (Tables 2, 4). Nonetheless, **1** appeared to exert slightly more efficient potentiation of antibiotics in *P. aeruginosa*. In contrast, **14** is four residues shorter than **15**, and hence exhibited a less pronounced effect on the viability of mammalian cells, since this effect is strongly dependent on oligomer length (Jahnsen et al., 2014). Nonetheless, shortening of oligomer length gives rise to a concomitantly lower antibacterial activity, as also seen in the MDR strains of *K. pneumoniae* and *P. aeruginosa* (Table 2), yet **14** retained a similar degree of synergy with antibiotics (Table 4). Consequently, the selectivity indexes for **14** in combination with rifampicin or azithromycin in *E. coli* proved to be the most favorable among all of the peptidomimetic-antibiotic combinations tested. However, due to the typically 2- to 8-fold higher concentrations of **14** in synergistic combinations, these gave rise to less pronounced selectivity for *K. pneumoniae* and *P. aeruginosa* over mammalian cells (as reflected in its SIs; Table 2). While subclass I and II peptidomimetics share the same α-peptide/β-peptoid hybrid backbone structure, subclass II is characterized by a mixed Lys and hArg content (in a 1:1 ratio) of cationic residues (Table 2). The increased effect of subclass II peptidomimetics **25** and **26** on the viability of mammalian cell lines may be correlated to the presence of hArg residues displaying guanidinium functionalities, which confer an increased propensity to interact with, and thereby disrupt, mammalian membranes (Chan et al., 2006; Hein-Kristensen et al., 2011; Findlay et al., 2012; Jahnsen et al., 2014). However, even though **28** belongs to subclass II it had a similarly low effect on mammalian cell viability as that of compounds **1** and **15**, which may be explained by its modification with a polar PEG-like moiety (i.e., 3,6,9-trioxadecanoyl; TODA) instead of a cationic moiety containing amine functionalities (i.e., NDab or spermine-acetyl moiety present in **25** and **26**, respectively). Thus, the increased effect of **25** and **26** on mammalian cell viability may be due to their increased net cationic charge (Jahnsen et al., 2015). Moreover, while **28** retained antibacterial potency similar to its parent unmodified compound, it had a lower effect on mammalian cell viability than the parent compound (for NIH 3T3: EC_50_ ~218 μM vs. 117 μM) (Jahnsen et al., 2015), suggesting that introduction of the TODA moiety is beneficial with respect to therapeutic utility.

Interestingly, nine compounds in the peptidomimetic array contained the subclass II core structure, yet only four compounds were detected as potentiators of antibiotics. Another shorter peptidomimetic (i.e., **10**) also potentiated rifampicin activity (but not that of azithromycin) in MDR *E. coli*. In contrast, the other five compounds (i.e., **9**, **12**, **13**, **27**, and **29**) did not exhibit any potentiation activity, detectable at the low concentrations tested in the screening (Table 1). This suggests that addition of a palmitoyl moiety (in **9**, **12**, **13**), a cinnamoyl moiety (in **27**), a fluorinated phenylalanine (in **29**), or shorter oligomers (i.e., **9** and **13**) may result in lowered potency or abolish potentiation, while similar modifications (e.g., in **29**) did not alter the antibacterial activity (Jahnsen et al., 2015). Based on these observations, there appears to be distinct physicochemical and/or overall structural requirements for achieving efficient potentiation of antibiotics and high direct antibacterial activity, respectively.

The present results infer that peptidomimetic **1** has a potential for repurposing rifampicin and azithromycin for treatment of MDR Gram-negative infections. While this peptidomimetic was considered to be the most promising due to its low toxicity (with ensuing favorable cell selectivity) and ability to exert synergy with antibiotics at submicromolar levels in all three species, the additional peptidomimetics with low effect on mammalian cell viability, characterized in this study, may also merit further investigation. Since a prerequisite for successful combination therapy is that the effective concentrations of both peptidomimetic and antibiotic can be achieved at the infection site, it is critical that future studies address *in vivo* pharmacokinetic/pharmacodynamics and acute toxicity to assess the clinical potential of such peptidomimetic-antibiotic combinations. In particular, co-formulation of antibiotic and peptidomimetics as nanoparticles would be preferable to ensure *in vivo* co-localization of the compounds in the appropriate ratios (Carmona-Ribeiro and de Melo Carrasco, 2014; Liu et al., 2016; Nordström and Malmsten, 2017). While *in vivo* data currently are lacking for the α-peptide/β-peptoid hybrids described in the present study, peptidomimetics (including the 12-mer, H-(NLys-Nspe-Nspe)_4_-NH_2_ and the pentamer C12K-2α_8_) displaying similar structural features such as a high content of both cationic and hydrophobic residues (aromatic or aliphatic) have previously been found to exhibit *in vivo* activity (antibacterial and antiparasitic, respectively) (Zaknoon et al., 2011; Czyzewski et al., 2016). Based on overall structural similarity, the peptidomimetics identified in the present study appear likely to possess physicochemical properties compatible with *in vivo* activity. Moreover, clinical breakpoints for rifampicin and azithromycin may be used to estimate the therapeutic potential of the antibiotics in these drug combinations. Although no clinical breakpoint points are available for these antibiotics in Enterobacteriaceae or *P. aeruginosa*, bactericidal efficacy of the **1**-rifampicin combination occurred at concentrations ranging from 0.06 to 2 μg/mL in time-kill assays, which is below the resistance breakpoint for rifampicin alone in *Staphylococcus* species (R ≥4 μg/mL) (Clinical and Laboratory Standards Institute, 2017). Similarly, at concentrations below the azithromycin resistance breakpoint for *Salmonella typhi* (R ≥32 μg/mL), the **1**-azithromycin combination exerted growth-inhibitory and bactericidal activity. Recent studies also indicate that antibacterial efficacy of azithromycin therapy against Gram-negative pathogens *in vivo* is considerably higher than implied by *in vitro* susceptibility testing in standard MHB media (Lin et al., 2015; Ersoy et al., 2017). Importantly, low concentrations (≤1 μM) of peptidomimetic **1** were able to induce susceptibility to azithromycin and rifampicin in most of the clinical isolates tested. Thus the low effect of **1** on viability of mammalian cells and its high potency in combination with antibiotics may facilitate circumvention of pharmacological obstacles frequently associated with antimicrobial peptides and peptidomimetics (Molchanova et al., 2017a).

## Author Contributions

KB, HF, and LG conceived and designed the study. KB, BJ, HF, and LG contributed to experimental design. KB, BJ, and AH performed experiments. HN provided cytotoxicity experiments and data. KB wrote the first draft of the manuscript. HF, LG, AH, HN, and KB wrote sections of the manuscript. All authors edited, revised, and approved the manuscript for submission.

### Conflict of Interest Statement

The authors declare that the research was conducted in the absence of any commercial or financial relationships that could be construed as a potential conflict of interest.
