# Supplementary material for: Repurposing Azithromycin and Rifampicin Against Gram-Negative Pathogens by Combination With Peptidomimetics
Source: Front Cell Infect Microbiol. 2019 Jul 2;9:236. doi: 10.3389/fcimb.2019.00236 (PMC6615261; doi:10.3389/fcimb.2019.00236)
Supplement: Supplementary file 1 [file Data_Sheet_1.PDF]

Supplementary Material

Repurposing Azithromycin and Rifampicin against Gram-Negative Pathogens by Combination with Peptidomimetics

Kristin R. Baker [1,2*], Bimal Jana [1,2], Anna Mette Hansen [3], Hanne Mørck Nielsen [4], Henrik Franzyk [3*], Luca Guardabassi [1,2]

*** Correspondence:**
Kristin R. Baker
kribake@sund.ku.dk
Henrik Franzyk
henrik.franzyk@sund.ku.dk

1 Supplementary Data

Table S1: Peptidomimetic sequences from Table 1 and molecular weights

Number in Screen	Compound sequence	MW (g/mol)
**1**	H-(Lys-βNPhe)$_8$-NH$_2$	3470.44
**2**	Ac-(hArg-βNPhe)$_8$-NH$_2$	3622.56
**3**	Ac-(hArg-βNPhe)$_5$-NH$_2$	2286.25
**4**	Ac-(hArg-βNspe)$_7$-NH$_2$	3275.31
**5**	Ac-(Lys-βNspe)$_8$-NH$_2$	3398.45
**6**	Ac-(Lys-βNspe)$_6$-NH$_2$	2563.6
**7**	Ac-(hArg-βNchx)$_6$-NH$_2$	2852.13
**8**	Ac-(hArg-βNchx-Lys-βNspe)$_3$-NH$_2$	2707.87
**9**	Pam-(Lys-βNspe-hArg-βNspe)-NH$_2$	1132.32
**10**	Ac-(Lys-βNspe-hArg-βNspe)$_2$-NH$_2$	1812.84
**11**	Pam-[(S)-Aoc]-(Lys-βNphe)$_6$-NH$_2$	2817.07
**12**	Pam-(Lys-βNspe-hArg-βNspe)$_3$-NH$_2$	2886.1
**13**	Pam-(Lys-βNspe-hArg-βNspe)$_2$-NH$_2$	2009.21
**14**	Ac-(Lys-βNPhe)$_6$-NH$_2$	2478.44
**15**	Ac-(Lys-βNPhe)$_8$-NH$_2$	3286.24
**16**	Ac-(hArg-βNspe)$_6$-(Trp)$_2$-NH$_2$	3188.26
**17**	Ac-(azaLys-βNspe-Lys-βNspe)$_4$-NH$_2$	3858.55
**18**	Ac-(bNLys-bNPhe)$_8$-NH$_2$	3398.45
**19**	Ac-(Lys-NTrp)$_8$-NH$_2$	3598.52

**20**	Ac-(NLys-Trp)$_8$-NH$_2$	3486.31
**21**	Ac-(bNLys-Trp)$_8$-NH$_2$	3598.52
**22**	Ac-(NLys-NPhe)$_8$-NH$_2$	3174.07
**23**	*p*-CF$_3$-benzoyl-(Lys-Phe)$_8$-NH$_2$	3304.14
**24**	Cin-(Lys-Phe)$_8$-NH$_2$	3262.18
**25**	NDab-Lys-βNspe-hArg-βNspe)$_3$-NH$_2$	2975.85
**26**	Spermine-(Lys-βNspe-hArg-βNspe)$_3$-NH$_2$	3346.14
**27**	Cin-(Lys-βNspe-hArg-βNspe)$_3$-NH$_2$	2777.83
**28**	TODA-(Lys-βNspe-hArg-βNspe)$_3$-NH$_2$	2807.85
**29**	F$_5$Ph-acetic acid-(5F-Phe)-(Lys-βNspe-hArg-βNspe)$_3$-NH$_2$	3092.9
**30**	VNW(NLys)$_2$VL(NLys)$_2$II(NLys)VA(NLys)-NH$_2$	2592.46
**31**	G(NLys)W(NLys)$_2$II(NLys)VA(NLys)-NH$_2$	2122.97
**32**	(NLys)LG(NLys)IW(NLys)I(Nlys)$_2$LF-NH$_2$	2056.04
**33**	(NLys)W(NLys)LF(NLys)$_2$VL(NLys)VLTTG-NH$_2$	2472.39
**34**	Pam-(Lys-βNspe)$_6$-NH$_2$	2759.98
**35**	Oct-(Lys-βNspe)$_6$-NH$_2$	2647.76
**36**	Pam-(Lys-βNPhe)$_6$-NH$_2$	2675.82
**37**	Lau-(Lys-βNPhe)$_6$-NH$_2$	2619.71
**38**	R(Tbt)R(hPhe)-NH$_2$	1187.21
**39**	R(Tbt)R(NPhe)	1173.18
**40**	Lau-Dap(Lau)-(Lys-βNspe-hArg-βNspe)$_3$-NH$_2$	3098.44
**41**	Ac-[(S)-Aoc]-(Lys-βNPhe)$_6$-NH$_2$	2620.7
**42**	Oct-[(S)-Aoc]-(Lys-βNphe)$_6$-NH$_2$	2704.86
